# Cryotherapy in the Management of Vital Pulp: A Case Report

**DOI:** 10.7759/cureus.61574

**Published:** 2024-06-03

**Authors:** Soumya Singhai, Shivkumar Mantri, Bonny Paul, Kavita Dube, Kushal P Taori

**Affiliations:** 1 Department of Conservative Dentistry and Endodontics, Hitkarini Dental College and Hospital, Jabalpur, IND; 2 Department of Orthodontics, Sharad Pawar Dental College, Datta Meghe Institute of Higher Education and Research, Wardha, IND

**Keywords:** biomaterial-based scaffolds, vital pulp therapy, reversible pulpitis, indirect pulp capping, direct pulp capping, cryotherapy

## Abstract

Cryotherapy in vital pulp treatment is a procedure that involves the use of extreme cold temperatures to manage inflammation and promote healing in the dental pulp tissue. It has shown potential in preserving pulp vitality and reducing post-operative discomfort in procedures such as partial and full pulpotomy. Vital pulp therapy (VPT) aims to preserve the vitality and function of the dental pulp. With the proper diagnosis, technique, and materials, it can effectively treat moderately inflamed pulp and minimize the need for more invasive procedures. This article presents a case of vital pulp cryotherapy in a patient having moderately inflamed pulp.

## Introduction

Cryotherapy derives from the word "Kr'yos," which means "cold" [1]. The purpose of cold application is to lower the temperature and eliminate heat of the healthy tissue. Applying cryotherapy to tissue reduces inflammation and edema, lowers hemorrhage, and slows down the conduction of the nerve [2]. Furthermore, cold application also demonstrates the reduced number of leukocytes attracting to the capillary walls resulting in a reduced number of cells migrating to the damaged or affected area. This process of reduced endothelial dysfunction lowers the inflammation of the tissue [3]. An additional advantage of cryotherapy application is a pharmacological intervention to minimize the use of local anesthetics or opioids. In different modalities of endodontic treatment, this cold application was proven to reduce post-operative pain.

Vital pulp therapy (VPT) is an endodontic procedure that aims to maintain and restore the vitality of the pulp tissue [4]. Clinically VPT is classified into two types: direct and indirect pulp capping. Pulp capping is a minimally invasive procedure that preserves the vitality of the pulp when the cause is a deep carious lesion or mechanical exposure to a normal pulp [5]. The exposed pulp-dentine tissue is capped with a medicament with the purpose of repair and regeneration and also to accelerate the process of dentine bridge formation. In the pulp capping procedure, calcium hydroxide causes necrosis of the pulp, the biggest disadvantage, and hence, is replaced by advanced materials such as bio dentine and mineral trioxide aggregate (MTA). Multiple variables can influence the outcome and indications for pulp vitality therapy such as choice of capping medicament, number of sites and exposure, pathological state of remaining pulp tissue, radiographic and clinical diagnosis, patient age, anatomic location of teeth, and bleeding time. Indications and contraindications for vital pulp cryotherapy are systemically healthy individuals presenting “extremely deep carious lesion” in a periodontally healthy tooth, responding within normal limits to the cold test, and not showing any rarefaction on the intraoral periapical radiograph. Teeth had a pre-treatment pulpal diagnosis of “reversible pulpitis” and an apical diagnosis of “normal apical tissues” was indicated. Endodontic patients whose pre-treatment diagnosis of the pulp tissue is necrosis, sinus (acute or chronic), apical abscess, periapical radiolucency, or teeth displaying an exaggerated or lingering response to cold testing are contraindicated. This article presents a case of vital pulp cryotherapy on tooth 46 having carious exposure to pulp.

## Case presentation

**Figure 1:**
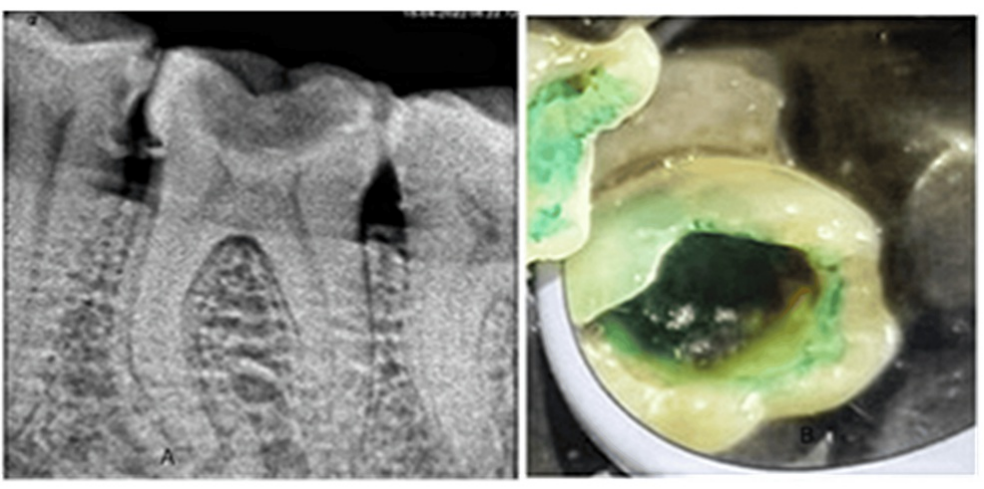
A shows pre-operative IOPA and B shows the application of caries indicator dye IOPA: intraoral periapical radiograph

A 35-year-old female patient reported with the chief complaint of food lodgment in a lower right molar tooth. The patient reported increased sensitivity to cold and sweet food, which would spontaneously subside. Clinical examination revealed a deep carious lesion on the occlusal surface. The tooth was not tender to percussion. The adjacent soft tissue appeared normal, and no swelling or sinus tracts were observed. Medical history was non-contributory. A digital intraoral periapical radiograph (IOPA) was obtained, and pulpal sensibility tests were performed (Figure 1).

**Figure 2:**
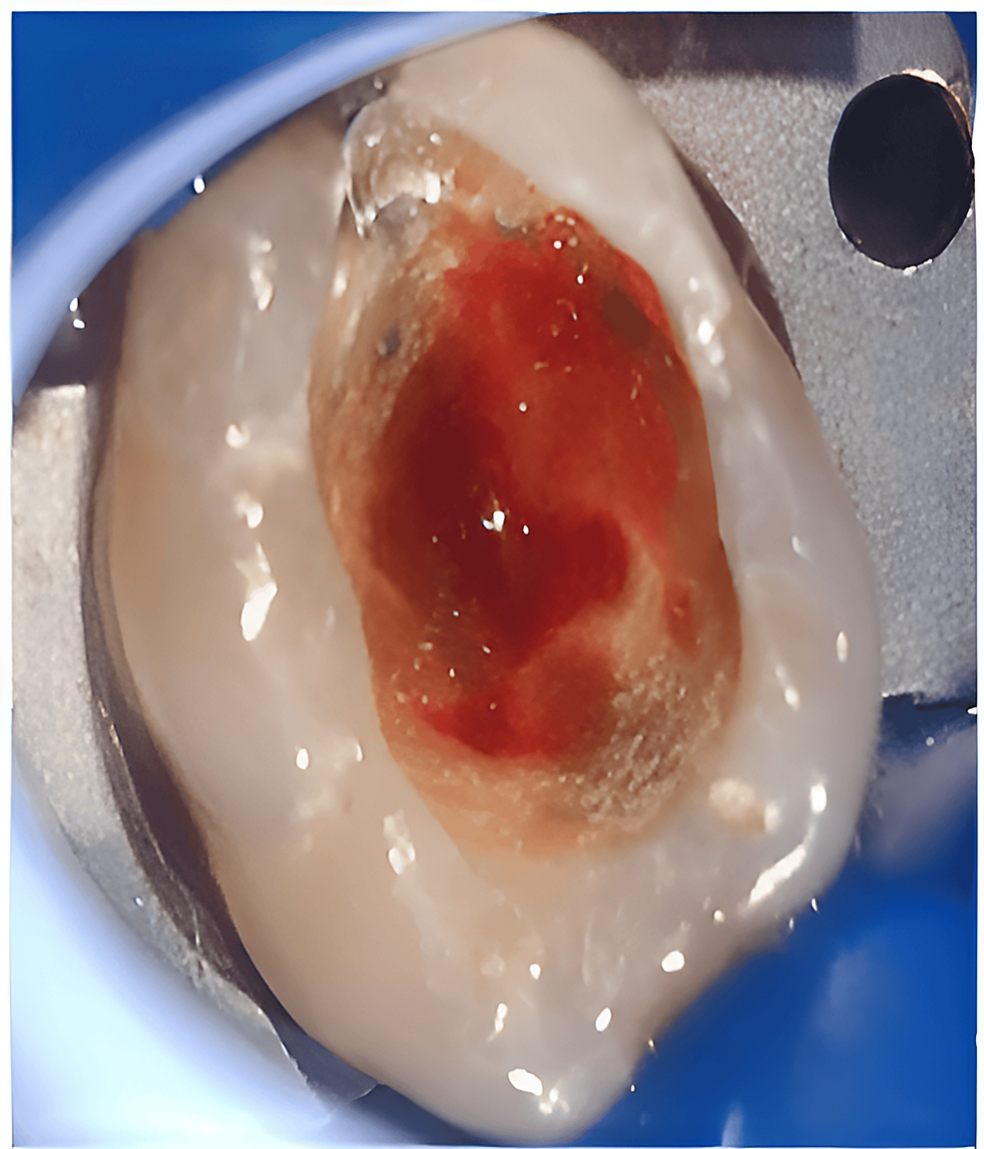
Pulpal tissue hemorrhaging

**Figure 3:**
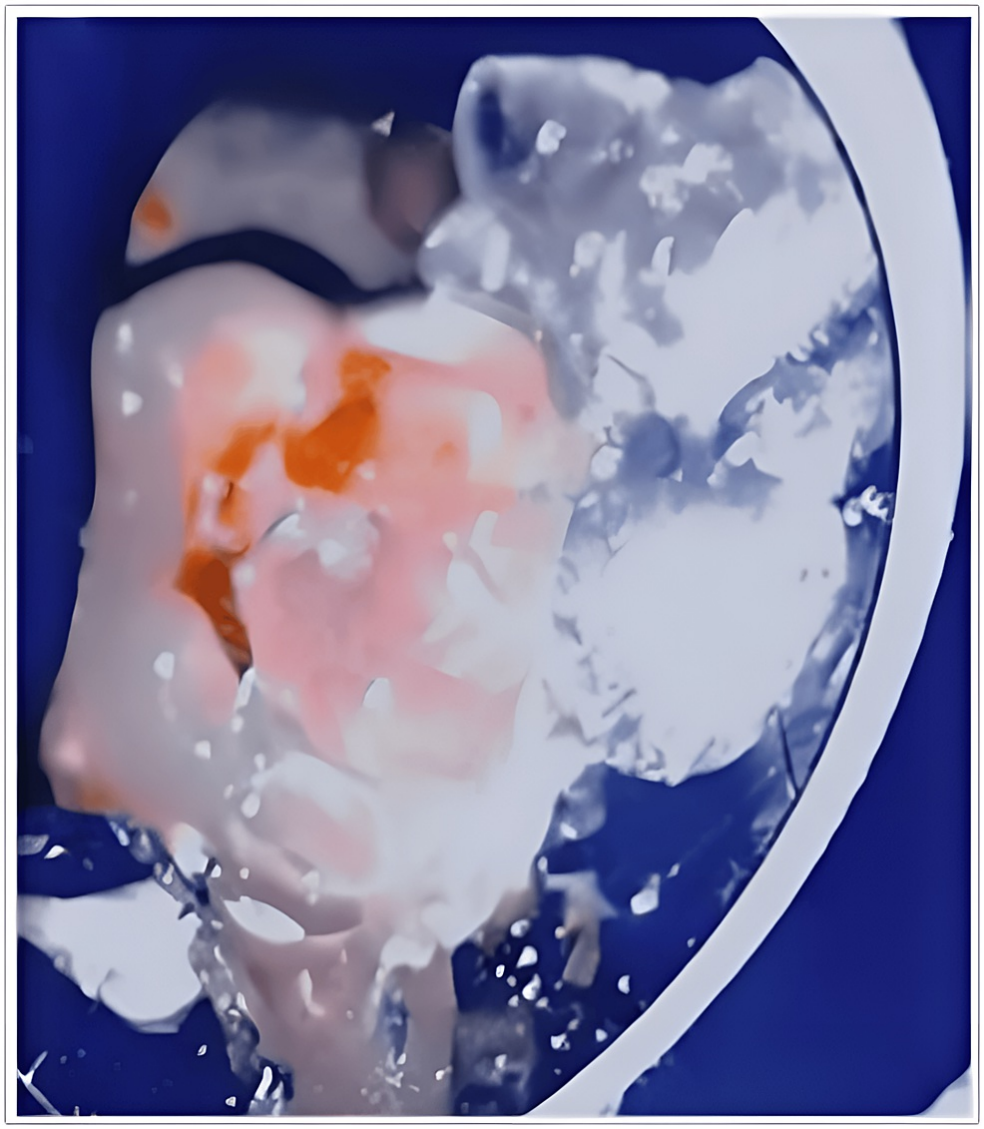
Application of sterile-water ice for cryotherapy

Cold test elicited a painful response, which lingered more than 10 seconds after the removal of the stimulus. Based on clinical and radiographic findings, the diagnosis of irreversible pulpitis associated with apical periodontitis was made. The patient was informed of the diagnosis and detailed therapeutic procedure including the importance of maintaining vitality, as the the survival of vital teeth is more compared to nonvital teeth. After obtaining informed consent, local anesthesia was administered. Pulpal anesthesia was confirmed with the subjective symptoms and objective tests. Rubber dam was applied and disinfected. Subsequent to carious tooth tissue removal, using high-speed bur and water spray coolant, access to pulp tissue was gained, and pulpotomy was carried out using a spoon excavator. As the inflamed pulp started bleeding (Figure 2), crushed sterile ice was placed over the exposed pulp tissue and the tooth (Figure 3).

**Figure 4:**
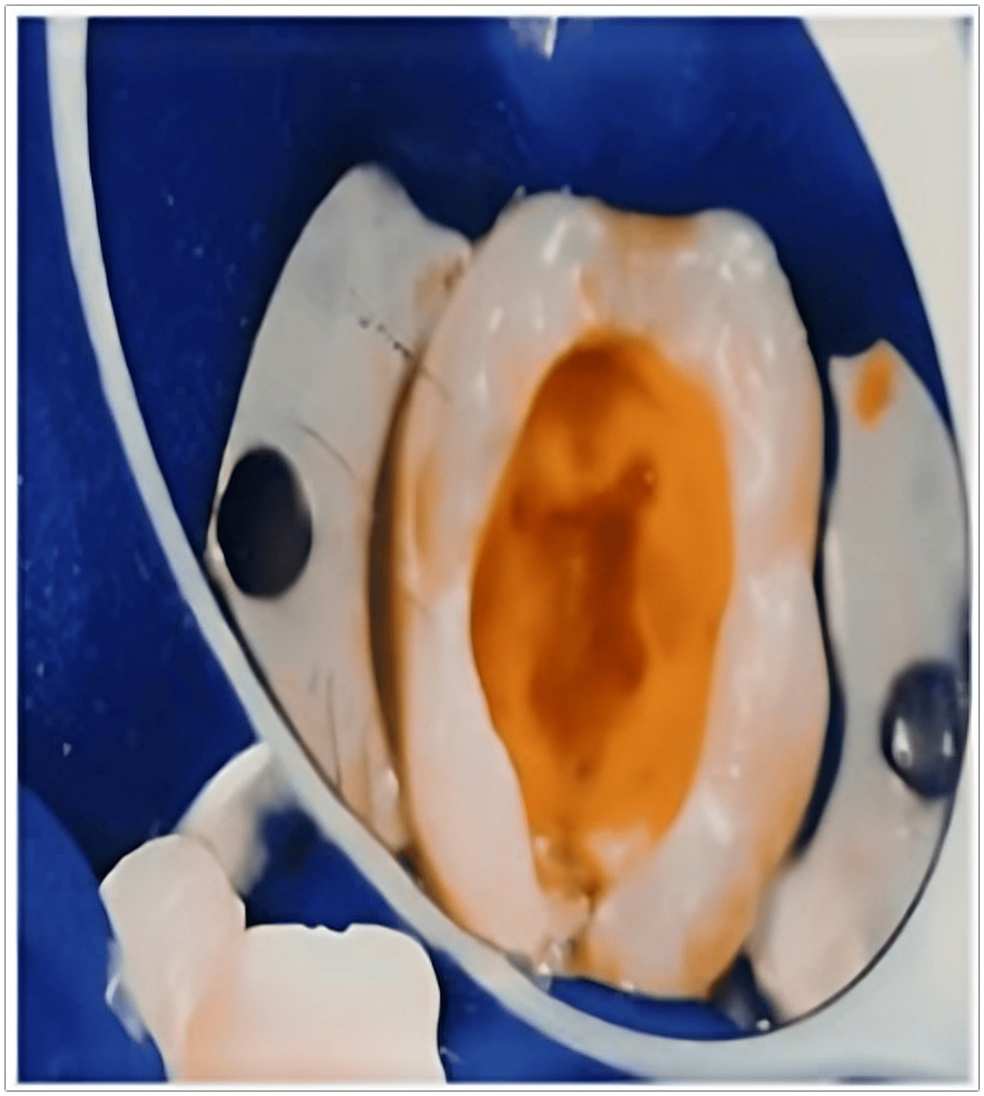
Application of 17% EDTA for one minute EDTA: ethylenediaminetetraacetic acid

After approximately 60 seconds, the melted ice was removed with high-speed suction. After achieving hemostasis, the cavity was irrigated with 17% ethylenediaminetetraacetic acid (EDTA) solution for 60 seconds and then with normal saline (Figure 4). 

**Figure 5:**
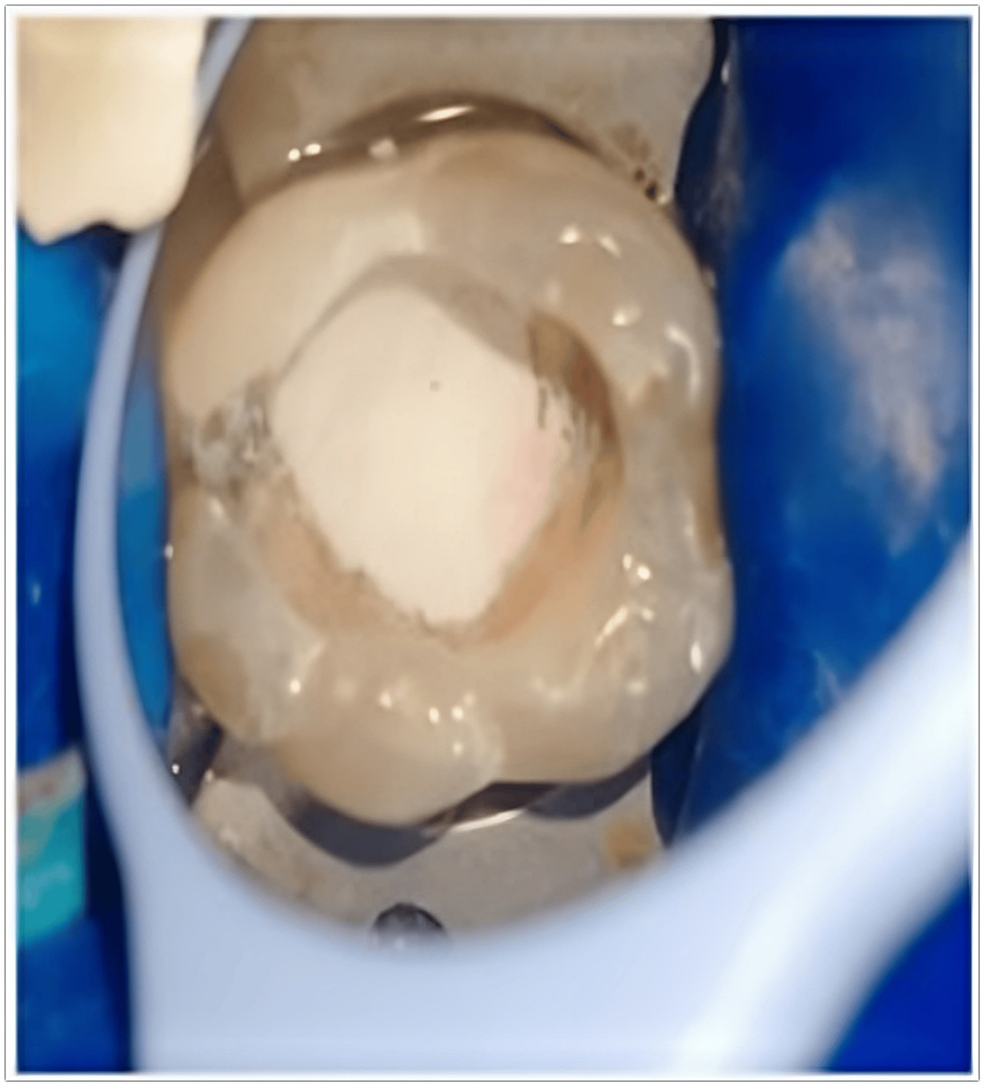
After achieving hemostasis, MTA was applied MTA: mineral trioxide aggregate

**Figure 6:**
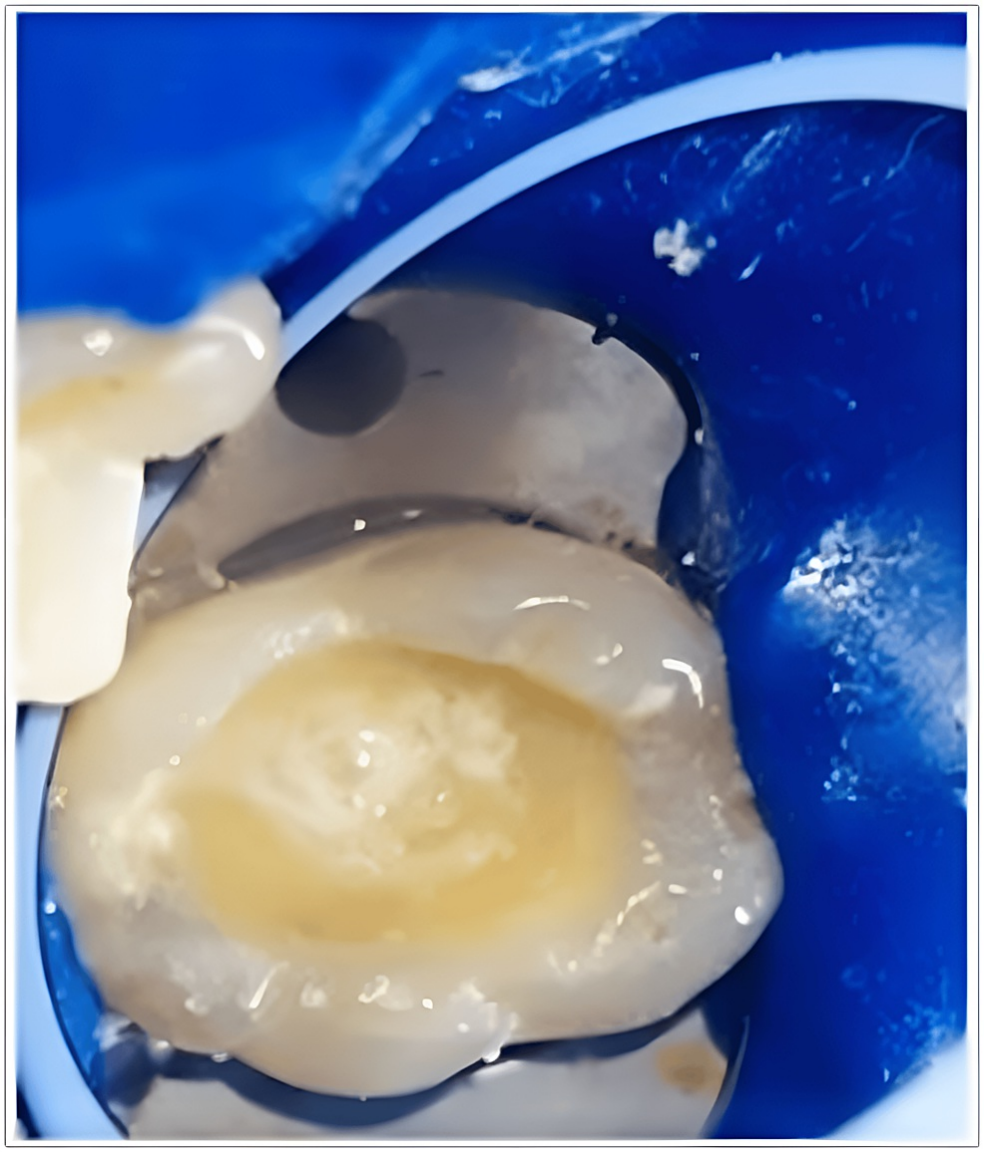
Application of RMGIC over bio-ceramic material RMGIC: resin-modified glass ionomer cement

**Figure 7:**
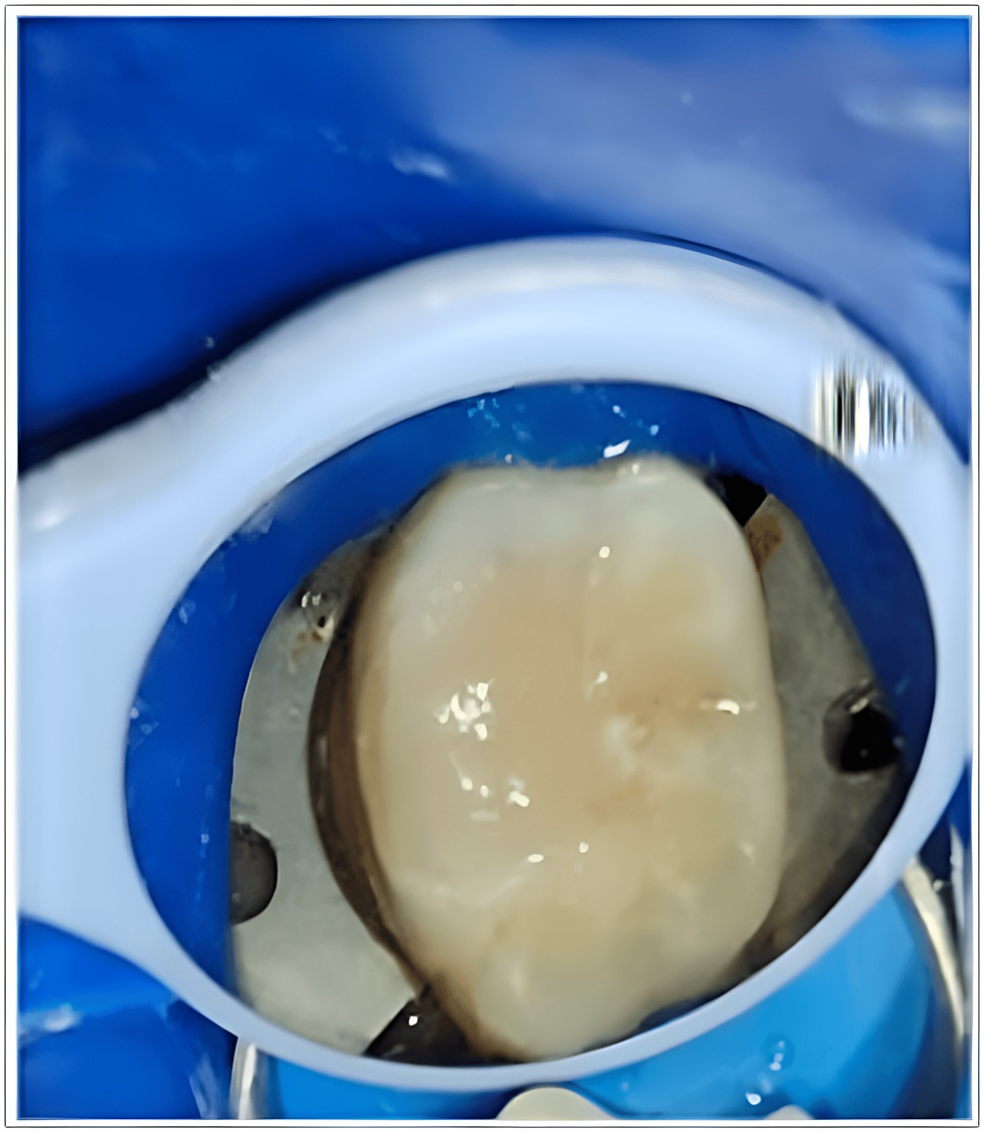
Final composite restoration

Bio-ceramic material MTA (Figure 5) was placed followed by light-cured glass ionomer cement (GIC) (Figure 6). The definitive final restoration was done using the nanohybrid composite (Figure 7).

**Figure 8:**
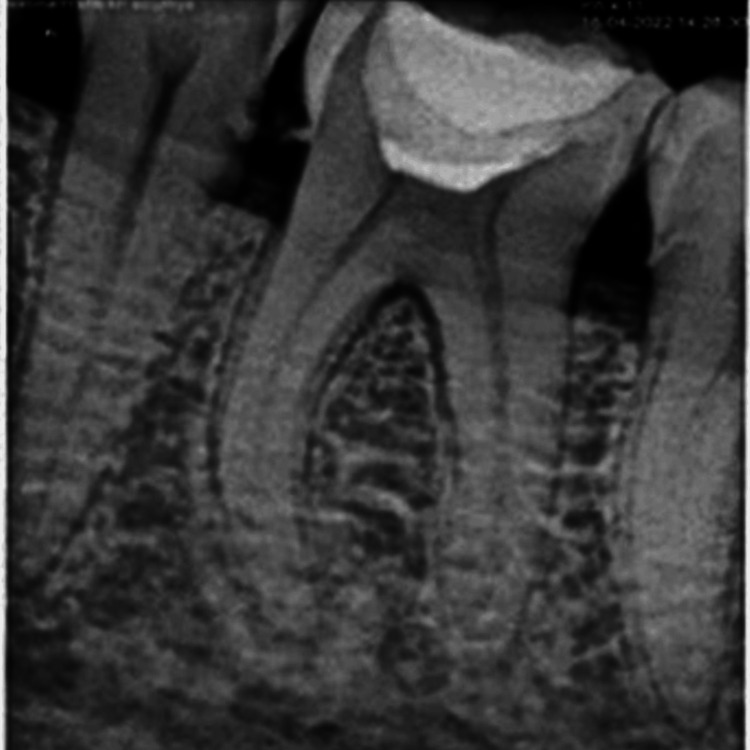
Immediate post-operative IOPA IOPA: intraoral periapical radiograph

Occlusion was checked, and a periapical radiograph was obtained. Analgesics were prescribed, and the patient was instructed to take them only in case of unbearable post-operative pain. The patient was advised to follow up after two weeks (Figure 8).

**Figure 9:**
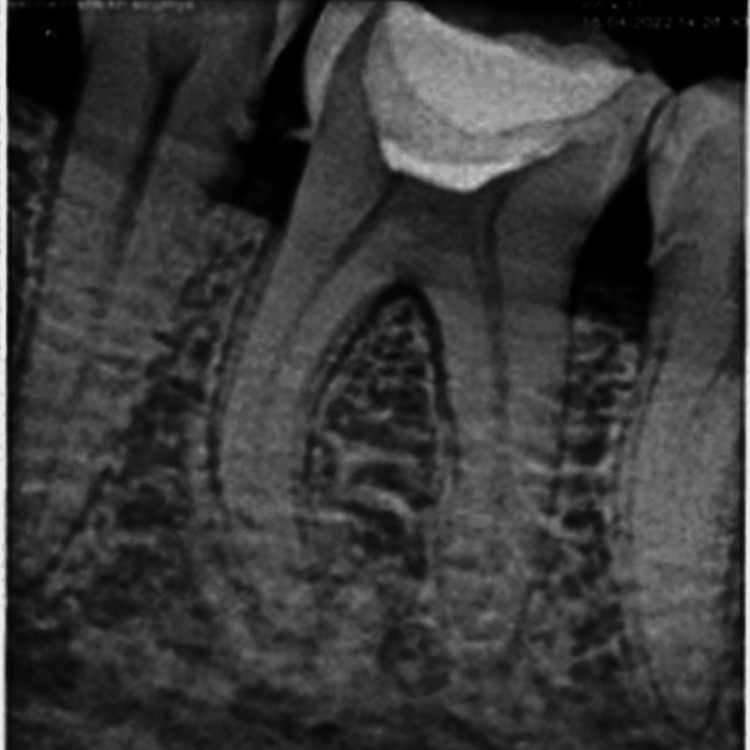
Recall radiograph after two weeks

At two weeks of recall, the patient was asymptomatic. She has not taken analgesic because of a lack of post-operative pain. The treated tooth was not tender to percussion and responded favorably to electric pulp testing indicating the vitality of remaining pulp. IOPA revealed no radiographic evidence of periapical deterioration. The patient was advised of yearly follow-up (Figure 9).

## Discussion

Reversible pulpitis, which is limited to coronal pulp, directly adjacent to the deep carious lesion, does not involve the entire radicular pulp and can be treated by partial or full pulpotomy. In cases of the diseased pulp, which continues to bleed, inflammation has extended apically where the entire radicular pulp is involved and then pulpectomy is considered a safe and effective alternative to treat the carious exposed pulp. In Ricucci et al., 15% of cases of symptomatic irreversible pulpitis become reversible pulpitis clinically and also concluded the correlation of clinical and histological match of reversible pulpitis to be 96% and irreversible pulpitis to be 85% [6].

Matsuo et al. discovered that pulpal bleeding is a clinical indicator of the severity of pulpal inflammation [7]. If the bleeding is controlled within 10 minutes then the procedure of partial pulpotomy should be considered, and if it exceeds and bleeding does not stop then full pulpectomy should be considered as treatment. The procedure of partial pulpotomy can be achieved by using a spoon excavator. The advantage of using an excavator is to minimize the heat and tissue stress caused by high-speed bur. In cases of diseased pulp, which continues to bleed, inflammation has extended apically. In cases where the entire radicular pulp is involved, then pulpectomy is considered a safe and effective alternative to treat the carious exposed pulp. In the present case, sodium hypochlorite was not used to control pulpal hemorrhage, as it destroys dental pulpal stem cells and reduces the release of dentinal growth factor [8]. We used cryotherapy along with EDTA, as it releases bioactive dentinal growth factors. Dentin conditioning with EDTA promotes dental pulp stem cell migration, adhesion, and differentiation. EDTA has antimicrobial effects on gram-negative and gram-positive bacteria, yeasts, and fungi. It also induces antioxidants and anti-inflammatory activities [9]. In this case, MTA was used as a pulp capping agent. When MTA came into direct contact with the cells, it stimulated the upregulation of key odontoblastic genes such as osteocalcin (OCN) and dentin sialoprotein (DSP). This indicates that direct interaction between cells and MTA is vital for encouraging pulp cell differentiation into odontoblast-like cells, which play a crucial role in forming dentin bridges. Additionally, MTA prompted an increase in vascular endothelial growth factor (VEGF) secretion when in direct contact with the cells [10].

Liun et al. in their study concluded that failures of VPT occur within the first two weeks after treatment [11]. The teeth remaining asymptomatic for two weeks remained vital and functional. If the patient returns following VPT (phase I treatment) with unresolved symptomatic irreversible pulpitis within one week with the signs of acute or chronic abscess, necrosis, and asymptomatic apical periodontitis, the clinician must reevaluate, and if needed conventional endodontic therapy should be performed (phase II treatment).

## Conclusions

Vital pulp cryotherapy is a safe and effective treatment option to treat cases of inflamed coronal pulp. The clinical relevance of cryotherapy in pulpitis provides three basic tissue responses: vasoconstriction, inhibition of neural receptors, and decreased metabolic activity, thus decreasing edema, pain, and inflammation. The combined effect of the decreased release of chemical mediators of pain and slower propagation of neural pain signals, thus, produces reductions in post-operative pain. Prospective cohort studies and clinical trials are warranted for better evidence and to strengthen its usefulness.
